# A copper complex of an unusual hy­droxy–carboxyl­ate ligand: [Cu(bpy)(C_4_H_4_O_6_)]

**DOI:** 10.1107/S2056989021001286

**Published:** 2021-02-19

**Authors:** Sen Gao, Frank R. Fronczek, Andrew W. Maverick

**Affiliations:** a Department of Chemistry, Louisiana State University, Baton Rouge, LA 70803, USA

**Keywords:** crystal structure, copper(II) complex, tartronate, square pyramidal, dimerization

## Abstract

A copper(II) complex, (2,2′-bi­pyridine-κ^2^ *N*,*N*′)[2-hy­droxy-2-(hy­droxy­methyl-κ*O*)propane­dioato-κ^2^ *O* ^1^,*O* ^3^]copper(II), [Cu(C_4_H_4_O_6_)(C_10_H_8_N_2_)], containing the unusual anionic chelating ligand 2-(hy­droxy­meth­yl)tartronate, has been synthesized. [Cu(bpy)_2_(NO_3_)](NO_3_) was mixed with ascorbic acid and Dabco (1,4-di­aza­bicyclo­[2.2.2]octa­ne) in DMF (dimethylformamide) solution in the presence of air to produce the title compound. The structure consists of square-pyramidal complexes that are joined by Cu⋯O contacts [2.703 (2) Å] into centrosymmetric dimers. The C_4_H_4_O_6_^2−^ ligand, which occupies three coordination sites at Cu, has previously been identified as an oxidation product of ascorbate ion.

## Abstract 2

A five-coordinate copper(II) complex with bpy and 2-(hy­droxy­meth­yl)tartronate ligands forms centrosymmetric dimers *via* Cu⋯O contacts [2.703 (2) Å].

## Chemical context   

Copper complexes have drawn recent attention owing to applications in redox reactions (Zubair *et al.*, 2019 ▸; Maity *et al.*, 2010 ▸; Wang *et al.*, 2006 ▸) and oxygen transport (Sheykhi *et al.*, 2018 ▸; Liu *et al.*, 2016 ▸; Tadsanaprasittipol *et al.*, 1998 ▸; Kato *et al.*, 2016 ▸). The 2,2′-bi­pyridine ligand has been used in a variety of supra­molecular architectures (Fei *et al.*, 2013 ▸; John *et al.*, 2004 ▸; Seco *et al.*, 2000 ▸; Barquín *et al.*, 2010 ▸; Yuan *et al.*, 2008 ▸).
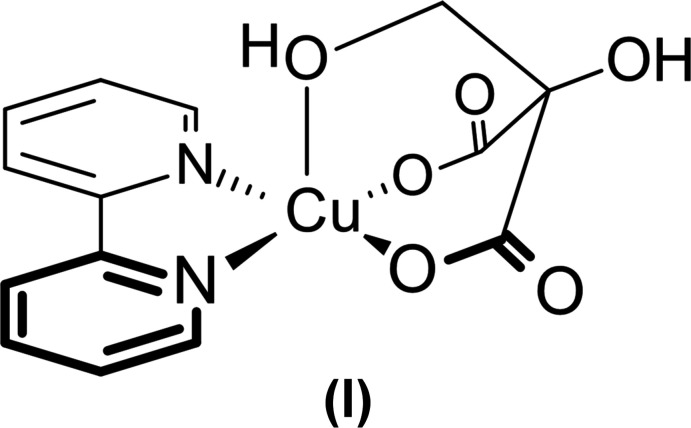


As a common reducing reagent, ascorbic acid has also been investigated in complex synthesis and redox reactions (Creutz, 1981 ▸; Niemelä, 1987 ▸; Sorouraddin *et al.*, 2000 ▸). For example, we have recently observed that mixtures of Cu complexes and ascorbate react with O_2_ to produce Cu^II^ oxalate complexes (Khamespanah *et al.*, 2021 ▸). However, to our knowledge, the particular degradation product of ascorbic acid observed here, 2-(hy­droxy­meth­yl)tartronic acid [2-(hy­droxy­meth­yl)-2-hy­droxy-1,3-propane­dioic acid], has been reported only a few times. It was identified by mass spectrometry as a product of oxidation of ascorbic acid (Niemelä, 1987 ▸; Löwendahl & Petersson, 1976 ▸) and two carbohydrates (Löwendahl *et al.*, 1975*a* ▸,*b* ▸). We have now isolated compound (I), a copper(II) complex of the 2-(hy­droxy­meth­yl)tartronate anion (see Scheme), and its crystal structure is reported here.

**Figure 1:**
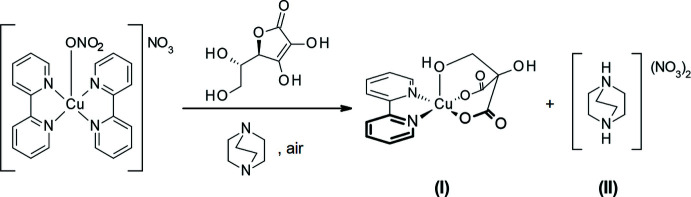
Preparation of the title compound, Cu(bpy)(C_4_H_4_O_6_) (I), with [DabcoH_2_](NO_3_)_2_ (II) as byproduct.

**Figure 2:**
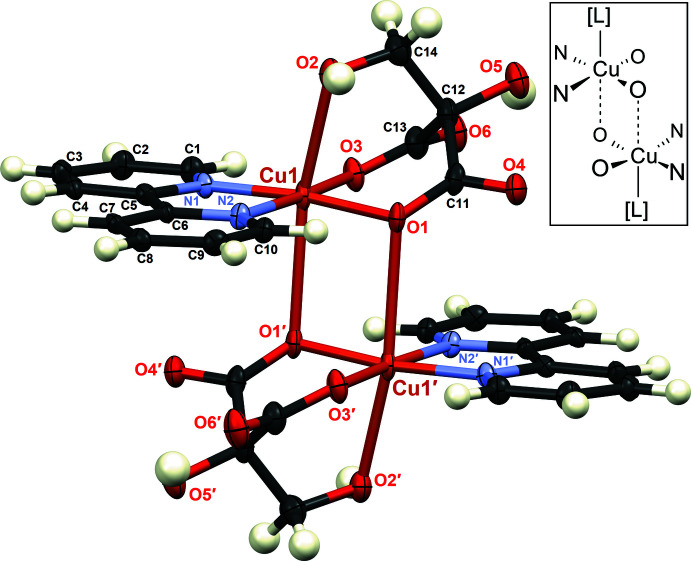
Crystal structure of (I). Ellipsoids are drawn at the 50% probability level; hydrogen atoms are displayed but not labeled. Primed and unprimed atoms are related by an inversion center, which brings the two square-pyramidal Cu(bpy)(C_4_H_4_O_6_) moieties into contact [Cu⋯O1′ = 2.703 (2) Å]. The inset is a schematic illustration of the dimerization.

The preparation of the title complex is shown in Fig. 1 ▸. A solution of [(bpy)_2_Cu(ONO_2_)]NO_3_ and Dabco (1,4-di­aza­bicyclo­[2.2.2]octa­ne) turned from blue to dark brown on addition of ascorbic acid, suggesting reduction of Cu^II^ to Cu^I^. The solution was then exposed to air. It turned green over a period of several days, and the title compound (I) could be crystallized (Fig. 2 ▸).

In this procedure, Dabco also crystallizes, in its doubly protonated form as colorless [DabcoH_2_](NO_3_)_2_ (II). We could not isolate the title compound (I) when Dabco was omitted from the reaction mixture. We determined the structure of (II) as well (Gao *et al.*, 2020 ▸). Although this structure was reported previously by Knope & Cahill (2007 ▸), the new structure provides improved resolution.

## Structural commentary   

The Cu atom in (I) adopts a square-pyramidal geometry, with coordination to two bpy N atoms and three O atoms from the 2-(hy­droxy­meth­yl)tartronate anion (C_4_H_4_O_6_^2–^).

The two inversion-related complexes in the unit cell make a dimer *via* two Cu⋯O contacts: Cu1⋯O1′ = 2.703 (2) Å. This kind of dimerization (see inset in Fig. 2 ▸) is commonly observed in 4- and 5-coordinate Cu^II^ complexes. It is discussed further in the *Database survey* section.

## Supra­molecular features   

**Figure 3:**
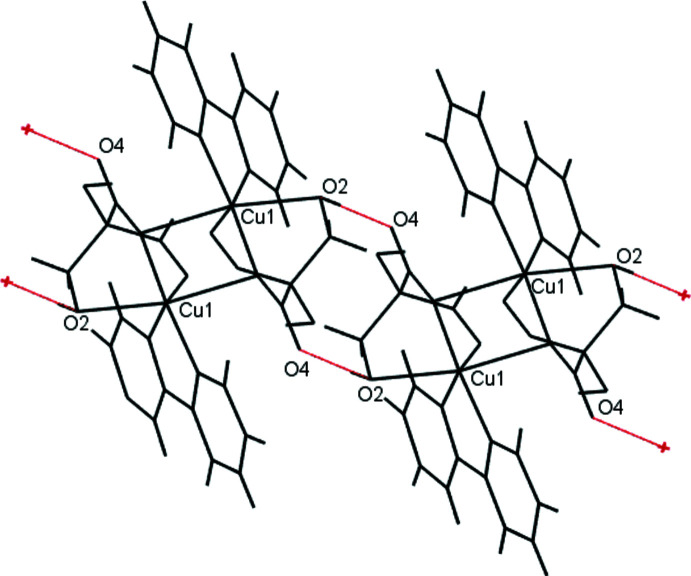
Packing structure of (I), showing the inter­molecular O2—H2*O*⋯O4 hydrogen bonds.

The structure of (I) includes two O—H⋯O hydrogen bonds, one intra­molecular and one inter­molecular; see Table 1 ▸. The inter­molecular hydrogen bonds form centrosymmetric hydrogen-bonded dimers with graph set 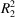
(12) (Etter *et al.*, 1990 ▸). These dimers are linked into chains in the [100] direction, as illustrated in Fig. 3 ▸.

## Database survey   

A survey of the Cambridge Structural Database (Version 5.40; Groom *et al.*, 2016 ▸) yielded four five-coordinate Cu^II^ complexes with 2,2′-bi­pyridine, one alcohol, and two carboxyl­ate ligands [CSD refcodes DAXVED (Antolini *et al.*, 1984 ▸), SEKXAI (Devereux *et al.*, 2006 ▸), TERTEQ (Ma *et al.*, 2006 ▸), and VAJTIL (Zhang *et al.*, 2010 ▸)]. The Cu atoms in these structures have a square-pyramidal geometry, with the alcohol ligand in the apical position, as in (I), with the following average angles and distances: N—Cu—N, 81.3 (10)°; Cu—N, 2.004 (13) Å; Cu—O(carboxyl­ate), 1.949 (15) Å; and Cu—O(alcohol), 2.32 (6) Å. These are similar to values in (I): N—Cu—N, 81.35 (9)°; Cu—N, 1.985 (2), 1.990 (2) Å; Cu—O, 1.9587 (19), 1.935 (2), and 2.384 (2) Å, respectively.

**Figure 4:**
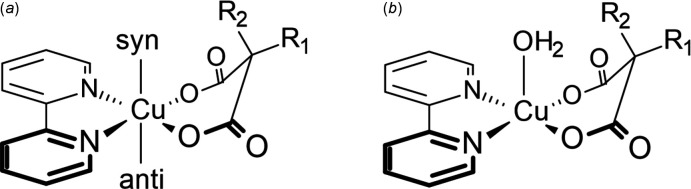
Generalized structures of [Cu(bpy)(malonate)] complexes: (*a*) showing the typical bending of the malonate ligand, with *syn* and *anti* coordination sites; (*b*) an example with H_2_O in the *syn* position, as can occur when *R*_2_ is small.

Another group of structures closely related to (I) is Cu(bpy)(malonate) (malonate = 1,3-propane­dioate); see Fig. 4 ▸. There are 14 such structures in the CSD, in all of which [as in (I)] the malonate C—O bonds are bent significantly out of the CuN_2_O_2_ coordination plane. Of these, seven [FIXDUM (Cui *et al.*, 2005 ▸), SAYCUQ (Gasque *et al.*, 1998 ▸), TIPZAT02 (Cernak, 2016 ▸), UNOJOY, UNOJUE, UNOKAL (Jaramillo-García *et al.*, 2016 ▸), and XECFOC (Manochitra *et al.*, 2012 ▸)] are monomeric, with *R*_2_ = H and syn H_2_O ligands [Fig. 4 ▸(*b*)]. This arrangement is similar to that observed in the Cu(bpy)(C_4_H_4_O_6_) moiety of (I), except that (I) contains an apical alcohol ligand rather than H_2_O. Because the alcohol in (I) is part of a small chelate ring, its coordination is bent slightly away from perpendicularity to the CuO_2_N_2_ plane [N1—Cu1—O2 104.04 (9), N2—Cu1—O2 91.77 (9)°]; the average N—Cu—OH_2_ angle in the above seven published structures is 93 (3)°.

In four structures [PUJJUC (Ghosh *et al.*, 2020 ▸), CIJNEQ (Dey *et al.*, 2013 ▸), MEHYON (Guan *et al.*, 1998*a* ▸,*b* ▸), and WAHVOR (Pasán *et al.*, 2004 ▸)], bulky *R*_2_ groups prevent *syn* coordination, and there are *anti* H_2_O ligands. In four structures [PUJJUC (Ghosh *et al.*, 2020 ▸), CELSIW01 (Reinoso *et al.*, 2007 ▸), CIJNEQ (Dey *et al.*, 2013 ▸), and PESBAR (Baldomá *et al.*, 2006 ▸)], dimers form as illustrated in Fig. 2 ▸, with Cu⋯O distances ranging from 2.315 (2) to 2.494 (3) Å. (Note: PUJJUC and CIJNEQ each contain two mol­ecules in the asymmetric unit, one a five-coordinate monomer and the other a dimer of four-coordinate complexes.) As far as we are aware, the present complex [Cu(bpy)(C_4_H_4_O_6_)] (I) is the only example of a Cu(bpy)(malonate) in which a five-coordinate species dimerizes. Our structure shows a considerably larger Cu⋯O distance in its dimers than the above four published examples. This is likely because of the apical alcohol ligand in (I): a five-coordinate species is less likely to form strong Cu⋯O associations than a four-coordinate species.

## Synthesis and crystallization   

**General procedures.** Reagents were used as received, from Sigma–Aldrich. FTIR spectra were recorded on a Bruker Tensor 27 spectrometer in attenuated total reflectance mode.

**Synthesis of Cu(bpy)(C_4_H_4_O_6_)**. To a mixture of [Cu(bpy)_2_(NO_3_)](NO_3_) (Marjani *et al.*, 2005 ▸) (25.5 mg, 0.075 mmol, in 2 mL of DMF) and Dabco (8.4 mg, 0.075 mmol, in 1 mL of DMF), ascorbic acid (13.2 mg, 0.075 mmol, in 1 mL of DMF) was added. The mixture turned to dark brownish-red. It was stirred for two days in air, during which time it turned green, and filtered. The filtrate was used for vapor diffusion with diethyl ether. Crystals of Cu(bpy)(C_4_H_4_O_6_) [(I), blue] and [DabcoH_2_](NO_3_)_2_ [(II), colorless] formed, which were suitable for X-ray analysis.

Cu(bpy)(C_4_H_4_O_6_). FTIR (cm^−1^) 3036*m*, 2853*w*, 1704*s*, 1667*m*, 1612*m*, 1412*m*, 1391*m*, 1362*m*, 1312*m*, 1204*s*, 1149*s*, 1055*m*, 1036*m*, 778*m*, 732*m*, 639*w*.

## Refinement   

Crystal data, data collection, and structure refinement are summarized in Table 2 ▸. All H atoms were visible in difference-Fourier maps. Coordinates of those on O were refined with O—H distances restrained to 0.88 (2) Å. Those on C were positioned geometrically (C—H = 0.95 Å for aromatic C, 0.99 Å for CH_2_) and treated as riding. Displacement parameters for H were assigned as *U*_eq_(H) = 1.2*U*_eq_(C) and 1.5*U*_eq_(O).

## Supplementary Material

Crystal structure: contains datablock(s) I. DOI: 10.1107/S2056989021001286/pk2655sup1.cif

Structure factors: contains datablock(s) I. DOI: 10.1107/S2056989021001286/pk2655Isup2.hkl

CCDC reference: 1966752

Additional supporting information:  crystallographic information; 3D view; checkCIF report

## Figures and Tables

**Table 1:** Hydrogen-bond geometry (Å, °)

*D*—H⋯*A*	*D*—H	H⋯*A*	*D*⋯*A*	*D*—H⋯*A*
O2—H2*O*⋯O4^i^	0.88 (2)	1.85 (2)	2.723 (3)	169 (4)
O5—H5*O*⋯O6	0.93 (2)	1.80 (3)	2.549 (3)	136 (3)

Symmetry code: (i) -x+2, -y+1, -z+1.

**Table 2:** Experimental details

Crystal data	
Chemical formula	[Cu(C_4_H_4_O_6_)(C_10_H_8_N_2_)]
*M*_r_	367.80
Crystal system, space group	Triclinic, *P*\overline{1}
Temperature (K)	90
*a*, *b*, *c* (Å)	7.6516 (5), 9.9272 (6), 10.0722 (6)
α, β, γ (°)	95.204 (4), 107.729 (4), 111.462 (4)
*V* (Å^3^)	660.34 (7)
*Z*	2
Radiation type	Mo *K*α
μ (mm^−1^)	1.69
Crystal size (mm)	0.15 × 0.09 × 0.07

Data collection	
Diffractometer	Bruker Kappa APEXII DUO CCD
Absorption correction	Multi-scan (*SADABS*; Krause *et al.*, 2015 ▸)
*T*_min_, *T*_max_	0.838, 0.891
No. of measured, independent and observed [*I* > 2σ(*I*)] reflections	18442, 4041, 2675
*R*_int_	0.063
(sin θ/λ)_max_ (Å^−1^)	0.715

Refinement	
*R*[*F* ^2^ > 2σ(*F* ^2^)], *wR*(*F* ^2^), *S*	0.049, 0.105, 1.02
No. of reflections	4041
No. of parameters	214
No. of restraints	2
H-atom treatment	H atoms treated by a mixture of independent and constrained refinement
Δρ_max_, Δρ_min_ (e Å^−3^)	0.74, −0.56

Computer programs: *APEX3* and *SAINT* (Bruker, 2016 ▸), *SHELXT2014/5* (Sheldrick, 2015*a* ▸), *SHELXL2014/7* (Sheldrick, 2015*b* ▸), *Mercury* (Macrae *et al.*, 2020 ▸), and *publCIF* (Westrip, 2010 ▸).

## supplementary crystallographic information

### Crystal data

**Table:**

[Cu(C_4_H_4_O_6_)(C_10_H_8_N_2_)]	*Z* = 2
*M**_r_* = 367.80	*F*(000) = 374
Triclinic, *P*1	*D*_x_ = 1.850 Mg m^−^^3^
*a* = 7.6516 (5) Å	Mo *K*α radiation, λ = 0.71073 Å
*b* = 9.9272 (6) Å	Cell parameters from 3354 reflections
*c* = 10.0722 (6) Å	θ = 2.2–29.3°
α = 95.204 (4)°	µ = 1.69 mm^−^^1^
β = 107.729 (4)°	*T* = 90 K
γ = 111.462 (4)°	Fragment, light blue
*V* = 660.34 (7) Å^3^	0.15 × 0.09 × 0.07 mm

### Data collection

**Table:**

Bruker Kappa APEXII DUO CCD diffractometer	4041 independent reflections
Radiation source: fine-focus sealed tube	2675 reflections with *I* > 2σ(*I*)
TRIUMPH curved graphite monochromator	*R*_int_ = 0.063
φ and ω scans	θ_max_ = 30.6°, θ_min_ = 2.2°
Absorption correction: multi-scan (SADABS; Krause *et al.*, 2015)	*h* = −10→10
*T*_min_ = 0.838, *T*_max_ = 0.891	*k* = −14→14
18442 measured reflections	*l* = −14→14

### Refinement

**Table:**

Refinement on *F*^2^	Primary atom site location: structure-invariant direct methods
Least-squares matrix: full	Secondary atom site location: difference Fourier map
*R*[*F*^2^ > 2σ(*F*^2^)] = 0.049	Hydrogen site location: mixed
*wR*(*F*^2^) = 0.105	H atoms treated by a mixture of independent and constrained refinement
*S* = 1.02	*w* = 1/[σ^2^(*F*_o_^2^) + (0.0436*P*)^2^ + 0.3982*P*] where *P* = (*F*_o_^2^ + 2*F*_c_^2^)/3
4041 reflections	(Δ/σ)_max_ < 0.001
214 parameters	Δρ_max_ = 0.74 e Å^−^^3^
2 restraints	Δρ_min_ = −0.56 e Å^−^^3^

### Special details

**Table:**

Geometry. All esds (except the esd in the dihedral angle between two l.s. planes) are estimated using the full covariance matrix. The cell esds are taken into account individually in the estimation of esds in distances, angles and torsion angles; correlations between esds in cell parameters are only used when they are defined by crystal symmetry. An approximate (isotropic) treatment of cell esds is used for estimating esds involving l.s. planes.

### Fractional atomic coordinates and isotropic or equivalent isotropic displacement parameters (Å^2^)

**Table:**

	*x*	*y*	*z*	*U*_iso_*/*U*_eq_	
Cu1	0.60628 (6)	0.63056 (4)	0.41304 (4)	0.01841 (12)	
O1	0.7261 (3)	0.5158 (2)	0.52799 (19)	0.0200 (5)	
O2	0.9188 (3)	0.7153 (3)	0.3763 (2)	0.0247 (5)	
H2O	0.931 (6)	0.635 (3)	0.346 (4)	0.037*	
O3	0.7221 (3)	0.7944 (2)	0.5778 (2)	0.0241 (5)	
O4	0.9916 (3)	0.5149 (2)	0.6990 (2)	0.0239 (5)	
O5	1.2089 (3)	0.8062 (3)	0.7594 (2)	0.0296 (5)	
H5O	1.178 (6)	0.869 (4)	0.813 (3)	0.044*	
O6	0.9741 (4)	0.9263 (3)	0.7840 (2)	0.0337 (6)	
N1	0.4537 (4)	0.7282 (3)	0.2933 (2)	0.0170 (5)	
N2	0.4699 (4)	0.4750 (3)	0.2317 (2)	0.0166 (5)	
C1	0.4522 (5)	0.8585 (3)	0.3373 (3)	0.0201 (6)	
H1	0.5308	0.9116	0.4337	0.024*	
C2	0.3410 (5)	0.9188 (3)	0.2483 (3)	0.0217 (7)	
H2	0.3409	1.0109	0.2833	0.026*	
C3	0.2293 (4)	0.8435 (3)	0.1071 (3)	0.0195 (6)	
H3	0.1527	0.8836	0.0433	0.023*	
C4	0.2309 (4)	0.7080 (3)	0.0597 (3)	0.0180 (6)	
H4	0.1567	0.6548	−0.0372	0.022*	
C5	0.3422 (4)	0.6519 (3)	0.1558 (3)	0.0142 (6)	
C6	0.3512 (4)	0.5078 (3)	0.1208 (3)	0.0142 (6)	
C7	0.2500 (4)	0.4119 (3)	−0.0127 (3)	0.0155 (6)	
H7	0.1676	0.4365	−0.0892	0.019*	
C8	0.2703 (4)	0.2792 (3)	−0.0335 (3)	0.0180 (6)	
H8	0.2000	0.2107	−0.1239	0.022*	
C9	0.3938 (5)	0.2480 (3)	0.0789 (3)	0.0191 (6)	
H9	0.4109	0.1583	0.0665	0.023*	
C10	0.4924 (4)	0.3482 (3)	0.2098 (3)	0.0180 (6)	
H10	0.5788	0.3267	0.2866	0.022*	
C11	0.9051 (5)	0.5800 (3)	0.6238 (3)	0.0217 (7)	
C12	1.0222 (5)	0.7493 (4)	0.6426 (3)	0.0247 (7)	
C13	0.8962 (5)	0.8297 (4)	0.6718 (3)	0.0258 (7)	
C14	1.0784 (5)	0.7878 (4)	0.5103 (3)	0.0284 (7)	
H14A	1.1263	0.8965	0.5195	0.034*	
H14B	1.1917	0.7614	0.5117	0.034*	

### Atomic displacement parameters (Å^2^)

**Table:**

	*U*^11^	*U*^22^	*U*^33^	*U*^12^	*U*^13^	*U*^23^
Cu1	0.0245 (2)	0.0195 (2)	0.00897 (15)	0.01379 (17)	−0.00134 (13)	−0.00131 (13)
O1	0.0255 (12)	0.0221 (12)	0.0104 (9)	0.0137 (10)	−0.0002 (8)	0.0018 (8)
O2	0.0272 (12)	0.0276 (13)	0.0172 (10)	0.0135 (11)	0.0035 (9)	0.0032 (9)
O3	0.0257 (12)	0.0268 (13)	0.0147 (10)	0.0143 (11)	−0.0012 (9)	−0.0040 (9)
O4	0.0247 (12)	0.0272 (12)	0.0202 (10)	0.0155 (10)	0.0031 (9)	0.0053 (9)
O5	0.0283 (13)	0.0337 (14)	0.0213 (11)	0.0169 (11)	−0.0006 (9)	−0.0024 (10)
O6	0.0366 (14)	0.0325 (14)	0.0221 (11)	0.0185 (12)	−0.0035 (10)	−0.0070 (10)
N1	0.0182 (13)	0.0181 (13)	0.0128 (10)	0.0084 (11)	0.0030 (9)	0.0005 (10)
N2	0.0212 (13)	0.0163 (13)	0.0129 (11)	0.0104 (11)	0.0045 (10)	0.0013 (10)
C1	0.0244 (17)	0.0168 (15)	0.0183 (13)	0.0108 (13)	0.0051 (12)	−0.0005 (12)
C2	0.0279 (18)	0.0163 (16)	0.0237 (15)	0.0138 (14)	0.0079 (13)	0.0037 (12)
C3	0.0195 (16)	0.0195 (16)	0.0223 (14)	0.0123 (13)	0.0052 (12)	0.0071 (12)
C4	0.0149 (15)	0.0242 (17)	0.0143 (13)	0.0095 (13)	0.0034 (11)	0.0031 (12)
C5	0.0136 (14)	0.0142 (14)	0.0145 (12)	0.0063 (12)	0.0046 (10)	0.0005 (11)
C6	0.0125 (14)	0.0167 (15)	0.0113 (12)	0.0060 (12)	0.0022 (10)	0.0019 (11)
C7	0.0116 (14)	0.0165 (15)	0.0140 (12)	0.0038 (12)	0.0020 (10)	0.0005 (11)
C8	0.0173 (15)	0.0170 (15)	0.0142 (12)	0.0039 (12)	0.0040 (11)	−0.0016 (11)
C9	0.0241 (16)	0.0142 (15)	0.0214 (14)	0.0101 (13)	0.0092 (12)	0.0022 (12)
C10	0.0202 (16)	0.0201 (16)	0.0155 (13)	0.0108 (13)	0.0056 (11)	0.0044 (12)
C11	0.0310 (18)	0.0229 (17)	0.0115 (12)	0.0158 (15)	0.0035 (12)	0.0006 (12)
C12	0.0257 (17)	0.0276 (18)	0.0162 (13)	0.0123 (15)	0.0010 (12)	0.0019 (13)
C13	0.0288 (18)	0.0260 (18)	0.0209 (15)	0.0149 (15)	0.0036 (13)	0.0015 (14)
C14	0.0271 (18)	0.0294 (19)	0.0235 (15)	0.0108 (16)	0.0049 (13)	0.0008 (14)

### Geometric parameters (Å, º)

**Table:**

Cu1—O3	1.935 (2)	C2—H2	0.9500
Cu1—O1	1.9587 (19)	C3—C4	1.391 (4)
Cu1—N1	1.985 (2)	C3—H3	0.9500
Cu1—N2	1.990 (2)	C4—C5	1.383 (4)
Cu1—O2	2.384 (2)	C4—H4	0.9500
O1—C11	1.288 (3)	C5—C6	1.474 (4)
O2—C14	1.417 (4)	C6—C7	1.381 (4)
O2—H2O	0.880 (18)	C7—C8	1.387 (4)
O3—C13	1.275 (4)	C7—H7	0.9500
O4—C11	1.236 (3)	C8—C9	1.377 (4)
O5—C12	1.417 (4)	C8—H8	0.9500
O5—H5O	0.929 (18)	C9—C10	1.380 (4)
O6—C13	1.236 (4)	C9—H9	0.9500
N1—C1	1.334 (4)	C10—H10	0.9500
N1—C5	1.355 (3)	C11—C12	1.549 (5)
N2—C10	1.339 (4)	C12—C13	1.532 (4)
N2—C6	1.358 (3)	C12—C14	1.558 (4)
C1—C2	1.375 (4)	C14—H14A	0.9900
C1—H1	0.9500	C14—H14B	0.9900
C2—C3	1.383 (4)		
			
O3—Cu1—O1	91.06 (8)	N1—C5—C6	114.2 (2)
O3—Cu1—N1	91.97 (9)	C4—C5—C6	124.4 (2)
O1—Cu1—N1	173.29 (10)	N2—C6—C7	121.5 (3)
O3—Cu1—N2	173.32 (9)	N2—C6—C5	114.4 (2)
O1—Cu1—N2	95.56 (9)	C7—C6—C5	124.1 (2)
N1—Cu1—N2	81.35 (9)	C6—C7—C8	119.1 (3)
O3—Cu1—O2	90.05 (9)	C6—C7—H7	120.4
O1—Cu1—O2	81.94 (8)	C8—C7—H7	120.4
N1—Cu1—O2	104.04 (9)	C9—C8—C7	119.0 (3)
N2—Cu1—O2	91.77 (9)	C9—C8—H8	120.5
C11—O1—Cu1	120.61 (19)	C7—C8—H8	120.5
C14—O2—Cu1	108.93 (18)	C8—C9—C10	119.5 (3)
C14—O2—H2O	106 (2)	C8—C9—H9	120.3
Cu1—O2—H2O	106 (2)	C10—C9—H9	120.3
C13—O3—Cu1	121.7 (2)	N2—C10—C9	121.9 (3)
C12—O5—H5O	96 (2)	N2—C10—H10	119.1
C1—N1—C5	119.1 (2)	C9—C10—H10	119.1
C1—N1—Cu1	125.71 (19)	O4—C11—O1	124.3 (3)
C5—N1—Cu1	115.18 (19)	O4—C11—C12	117.9 (3)
C10—N2—C6	119.0 (2)	O1—C11—C12	117.8 (2)
C10—N2—Cu1	126.08 (19)	O5—C12—C13	108.3 (2)
C6—N2—Cu1	114.81 (18)	O5—C12—C11	111.3 (2)
N1—C1—C2	122.5 (3)	C13—C12—C11	109.2 (3)
N1—C1—H1	118.8	O5—C12—C14	105.0 (3)
C2—C1—H1	118.8	C13—C12—C14	110.5 (3)
C1—C2—C3	119.0 (3)	C11—C12—C14	112.4 (2)
C1—C2—H2	120.5	O6—C13—O3	125.2 (3)
C3—C2—H2	120.5	O6—C13—C12	117.0 (3)
C2—C3—C4	119.0 (3)	O3—C13—C12	117.8 (3)
C2—C3—H3	120.5	O2—C14—C12	114.7 (3)
C4—C3—H3	120.5	O2—C14—H14A	108.6
C5—C4—C3	119.0 (3)	C12—C14—H14A	108.6
C5—C4—H4	120.5	O2—C14—H14B	108.6
C3—C4—H4	120.5	C12—C14—H14B	108.6
N1—C5—C4	121.4 (3)	H14A—C14—H14B	107.6
			
C5—N1—C1—C2	0.1 (5)	C6—N2—C10—C9	−2.0 (4)
Cu1—N1—C1—C2	−179.4 (2)	Cu1—N2—C10—C9	−178.8 (2)
N1—C1—C2—C3	−1.3 (5)	C8—C9—C10—N2	0.8 (5)
C1—C2—C3—C4	0.8 (5)	Cu1—O1—C11—O4	−179.0 (2)
C2—C3—C4—C5	0.8 (4)	Cu1—O1—C11—C12	−1.0 (4)
C1—N1—C5—C4	1.5 (4)	O4—C11—C12—O5	−6.9 (4)
Cu1—N1—C5—C4	−178.9 (2)	O1—C11—C12—O5	175.0 (2)
C1—N1—C5—C6	−178.4 (3)	O4—C11—C12—C13	−126.3 (3)
Cu1—N1—C5—C6	1.2 (3)	O1—C11—C12—C13	55.6 (3)
C3—C4—C5—N1	−2.0 (4)	O4—C11—C12—C14	110.7 (3)
C3—C4—C5—C6	177.9 (3)	O1—C11—C12—C14	−67.4 (4)
C10—N2—C6—C7	1.6 (4)	Cu1—O3—C13—O6	−175.1 (3)
Cu1—N2—C6—C7	178.7 (2)	Cu1—O3—C13—C12	6.5 (4)
C10—N2—C6—C5	−178.2 (3)	O5—C12—C13—O6	1.0 (4)
Cu1—N2—C6—C5	−1.1 (3)	C11—C12—C13—O6	122.3 (3)
N1—C5—C6—N2	−0.1 (4)	C14—C12—C13—O6	−113.5 (3)
C4—C5—C6—N2	−179.9 (3)	O5—C12—C13—O3	179.6 (3)
N1—C5—C6—C7	−179.9 (3)	C11—C12—C13—O3	−59.1 (4)
C4—C5—C6—C7	0.3 (5)	C14—C12—C13—O3	65.1 (4)
N2—C6—C7—C8	0.0 (4)	Cu1—O2—C14—C12	19.3 (3)
C5—C6—C7—C8	179.8 (3)	O5—C12—C14—O2	167.8 (3)
C6—C7—C8—C9	−1.2 (4)	C13—C12—C14—O2	−75.7 (3)
C7—C8—C9—C10	0.8 (4)	C11—C12—C14—O2	46.6 (4)

### Hydrogen-bond geometry (Å, º)

**Table:**

*D*—H···*A*	*D*—H	H···*A*	*D*···*A*	*D*—H···*A*
O2—H2*O*···O4^i^	0.88 (2)	1.85 (2)	2.723 (3)	169 (4)
O5—H5*O*···O6	0.93 (2)	1.80 (3)	2.549 (3)	136 (3)

Symmetry code: (i) −*x*+2, −*y*+1, −*z*+1.
